# The Bradford Infirmary Project

**Published:** 1914-02-14

**Authors:** 


### The Bradford Infirmary Project.

### TEXT OF THE RECOMMENDATIONS.

As we note in our second leading article this week the project by which the Corporation is to acquire the present buildings and site of the Bradford Royal Infirmary in return for representation on the Board to the extent of one-fourth of its membership was mooted last summer when a joint committee representing the City Council, the Insurance Committee, and the Infirmary Board discussed the matter. A sub-committee was formed, consisting of three members of each body, and the sub-committee agreed that the Infirmary representatives should report to the Board and then put forward a resolution embodying their recommendations. For the sake of the importance and novelty of the proposal we here append their recommendations in full:

That this Board offer to the City Council a representation of six members (out of twenty-four) on the board of management of the new infirmary, on condition that the City Council purchase the existing Royal Infirmary buildings and site for the sum of £100,000.

The Board consider that by such action the purely voluntary system of the Royal Infirmary would be safeguarded, and feel confident that if the above scheme be carried out an adequate annual income would be forthcoming to maintain the new infirmary by voluntary contributions.

The Corporation representatives expressed the opinion that the other hospitals in the City should be associated in the scheme and their recommendation slightly modified was adopted by the Bradford City Council as follows, on the 10th inst.: —

That the site of the Bradford Royal Infirmary, including all buildings and land, be acquired by the Corporation, and that such price be paid, having regard to these purposes, as shall be fixed by a competent valuer, subject to the approval of the Local Government Board to the borrowing of the purchase price; and further, that if such price, when fixed, shall be less than £100,000, the Corporation be recommended to apply to the Local Government Board for borrowing powers for the difference between the purchase price and £100,000 for the purpose of enabling the Corporation to enter into an agreement with the Infirmary Management under the Public Health Act, 1875, to pay over such difference as a lump sum towards the provision of new Infirmary accommodation in consideration of the reception at the new institution of the sick inhabitants of the City, the Corporation having representation on the Board of Management to the extent of one-fourth of the total number of members of the Board, and subject to such other terms and conditions of agreement as may be necessary or as the Local Government Board may impose.

